# Supplementary material for: How long is enough to detect terrestrial animals? Estimating the minimum trapping effort on camera traps
Source: PeerJ. 2014 May 8;2:e374. doi: 10.7717/peerj.374 (PMC4017883; doi:10.7717/peerj.374)
Supplement: Supplemental Information 2 [file peerj-02-374-s002.pdf]

古田山国家级自然保护区野外实验批准书

批准号：GTS2006005

兹有浙江大学生命科学学院生态研究所科研人员在浙江古田山国家级自然保护区开展动植物科学研究，其野外调查及相关科研活动符合《中华人民共和国自然保护区条例》（1994 年 10 月 9 日）和《中华人民共和国野生动物保护法》（2004 年 8 月 28 日）等相关法律法规的规定，现准予在本保护区开展科学研究。

开化古田山国家级自然保护区管理局

2006 年 3 月 1 日

地址：浙江省开化县城关镇芹北路 41 号 邮编：324300 电话：0570-6017208 传真：0570-6017208
